# Calcium-Sensing Receptor in Human Peripheral Blood T Lymphocytes Is Involved in the AMI Onset and Progression through the NF-κB Signaling Pathway

**DOI:** 10.3390/ijms17091397

**Published:** 2016-08-24

**Authors:** Jing-Ya Zeng, Jing-Jing Du, Ying Pan, Jian Wu, Hai-Liang Bi, Bao-Hong Cui, Tai-Yu Zhai, Yong Sun, Yi-Hua Sun

**Affiliations:** 1 Department of Clinical Laboratory, Harbin Medical University Cancer Hospital, Harbin 150086, China; zengjingya121@163.com (J.-Y.Z.); 13019019570@163.com (Y.P.); 18346144845@163.com (H.-L.B.); cuibaohongsun@163.com (B.-H.C.); zhaitaiyu0810@163.com (T.-Y.Z.); 2 Blood Transfusion Department, the First Affiliated Hospital of Harbin Medical University, Harbin 150086, China; 18745793906@126.com; 3 Department of Cardiology, the Second Affiliated Hospital of Harbin Medical University, Harbin 150086, China; wujian780805@163.com

**Keywords:** calcium-sensing receptor, signaling pathway, lymphocyte, acute myocardial infarction, cytokine

## Abstract

Acute myocardial infarction (AMI) is a condition triggered by an inflammatory process that seriously affects human health. Calcium-sensing receptor (CaSR) in T lymphocytes is involved during the inflammation reaction. However, the relationship between them is not very clear. In this study, we collected human peripheral blood T lymphocytes from patients with AMI and in different stages of percutaneous coronary intervention (PCI) (at the onset of AMI, the first day after PCI (PCI-1), PCI-3, and PCI-5) to study the CaSR and NF-κB pathway protein expression, cytokine release and T cell apoptosis. The results showed that the expressions of CaSR, P-p65, Caspase-12, and the secretions of Th-1 and Th-2 type cytokines were increased at the onset of AMI, especially on the PCI-1. Meanwhile, the apoptosis rate of CD^3+^, CD^4+^ and CD^8+^ T lymphocytes also increased. However, from PCI-3, all the indicators began to decline. In addition, we also found that positive CaSR small interfering RNA (siRNA) transfection in T lymphocytes and NF-κB pathway blocker Bay-11-7082 reversed the increased expressions of CaSR, P-p65, Caspase-12, reduced the secretions of Th-1 and Th-2 type cytokines, and decreased T lymphocytes apoptosis rate not only in the AMI patients but also in the normal controls. All of these results indicated that CaSR in the human peripheral blood T lymphocytes were involved in the AMI onset and progression, which probably was related to the NF-κB pathway. Our study demonstrated the relationship between AMI and CaSR, and will provide new effective prevention theory and new targets for drug treatment.

## 1. Introduction

Acute myocardial infarction (AMI) is a complex multifactor pathogenic process, which often occurs on the basis of coronary atherosclerosis, and inflammatory reaction is an important factor in the pathogenesis of atherosclerosis. In recent years, percutaneous coronary intervention (PCI) has become a life-saving method for patients with AMI, which can narrow the infarct size and reduce the complications. However, some complications result from the recurrence risk, such as sudden death, arrhythmia and congestive heart failure, frequently occurring in AMI survival after PCI, which may be related to myocardial ischemia/reperfusion (IR) injury during PCI [1]. Myocardial IR injury is a complex process concerned with the imbalance of immune response and inflammatory reaction. The mechanism research and preventive measures are still unsatisfactory. A recent study in humans showed that AMI is associated with Th1/Th2 cytokine imbalance and some inflammatory cytokines are predictors for cardiovascular outcomes [2,3]. The inflammatory factors and cytokines are mainly released by lymphocytes. Additionally, extensive lymphocyte apoptosis occurred in the circulatory system during AMI injury [4]. It is increasingly being recognized that lymphocytes play a vital role in AMI injury [5]. Apoptosis of lymphocytes during an early stage of AMI is the major reason for inflammatory mechanism. It has also been proved that a reduction in lymphocyte apoptosis was associated with an improvement in the survival ratio in AMI [6]. 

Many membrane receptors can activate immuno-responsive cells, accompanying with various sequential kinase cascades activation. Calcium-sensing receptor (CaSR) and lymphocytes play a pathophysiological role through signal transduction pathways.

Calcium-sensing receptor (CaSR) is a member of the G protein-coupled receptor superfamily that exists in many tissues and cells. CaSR plays an important role in cell differentiation, proliferation, apoptosis, hormone secretion, and so on [7,8,9]. In a previous study, Sun et al. had proved that CaSR was involved in the rat cardiac IR injury combined with transient receptor potential channel [10]. In addition, Li et al. found that the activation of CaSR in normal human peripheral blood T lymphocytes could promote the secretions of IL-6 and TNF-α through MAPK and NF-κB pathways [11]. Wu et al. also indicated that CaSR in rat sepsis peripheral blood T lymphocytes influence lymphocytes to release cytokines and lymphocyte apoptosis [12,13]. However, there is no evidence to make certain the role of CaSR in human T lymphocytes on AMI occurrence and development. Thus, in this study, we have observed the CaSR role in human peripheral blood T lymphocytes at the onset of AMI and PCI postoperative. In addition, we found that CaSR in human T lymphocytes affected the T cells apoptosis and cytokines secretion involved in NF-κB pathways in AMI. This would provide new effective prevention and treatment theory for AMI. 

## 2. Results

### 2.1. Protein Expression of Calcium-Sensing Receptor (CaSR) in the T Lymphocytes at the Acute Myocardial Infarction (AMI) Onset and after Percutaneous Coronary Intervention (PCI)

**Figure 1:**
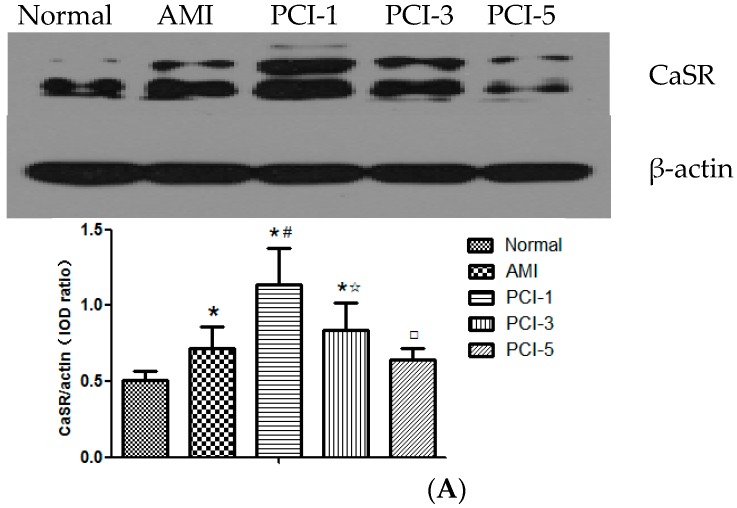

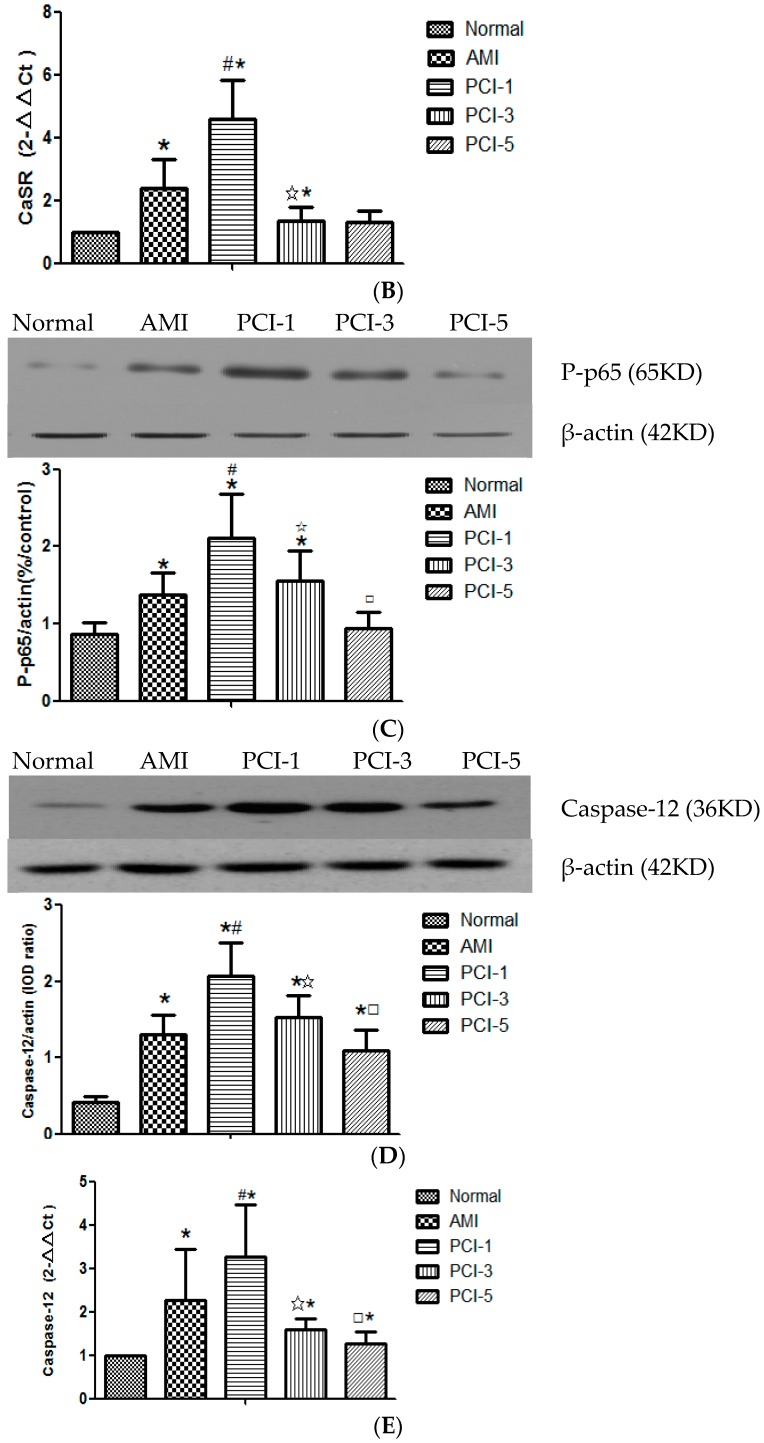
Protein and mRNA expressions of calcium-sensing receptor (CaSR) (**A**,**B**) and Caspase12 (**D**,**E**) in T lymphocytes and the phosphorylation level of NF-κB pathway protein (**C**) during the different stages in acute myocardial infarction (AMI). Protein expressions in T lymphocytes were tested by Western blot and qRT-PCR quantitative analysis (*n* = 20). Expression protein was quantified by densitometry. Expression results are representative of five experiments. * *p* < 0.05 vs. normal group; ^#^ *p* < 0.05 vs. AMI group; ^☆^ *p* < 0.05 vs. PCI-1 group; ^□^ *p* < 0.05 vs. PCI-3 group.

Western blotting detected two relative molecular mass of 120–170 KDa and 90–110 KDa of the CaSR protein. qRT-PCR quantitative analysis was used to detected the CaSR mRNA expression. From the results, it can find that both the CaSR protein and mRNA in T cells increased at the AMI onset and PCI-1 compared normal group, especially in the PCI-1 (*p* < 0.05), then began to descend from PCI-3. The CaSR mRNA level descended more quickly than CaSR protein. (Figure 1A,B).

### 2.2. Detection of the Pathway Protein at the AMI Onset and after PCI

Some researches had indicated that the CaSR activation triggers cytokines production from immuno-responsive cells through the NF-κB pathway [12]. In this study, in order to make clear whether NF-κB pathway was involved in AMI, we detected the phosphorylation of NF-κB pathway protein p65 by the Western blot. The results showed that the phosphorylation level of p65 increased at the onset of AMI and climbed to the summit in PCI-1. The level remained higher in PCI-3, then decreased to the normal level in PCI-5 (Figure 1C).

### 2.3. Expression of Caspase-12 at the AMI Onset and after PCI

Caspases are enzymes belonging to the group of cysteine proteases. These proteins are actively involved in inflammatory processes and apoptosis. Increase in the intracellular concentration of calcium can also stimulate the release of AIF from the mitochondrium and caspase-12 from the endoplasmic reticulum in caspase-independent apoptosis process [14]. From the obtained results of the WB and qRT-PCR quantitative analysis, we can find that the Caspase-12 in T cells kept on a higher level from AMI onset to PCI-5, especially in the PCI-1 (*p* < 0.05) (Figure 1D,E).

### 2.4. T-Lymphocyte Apoptosis at the AMI Onset and after PCI

**Figure 2:**
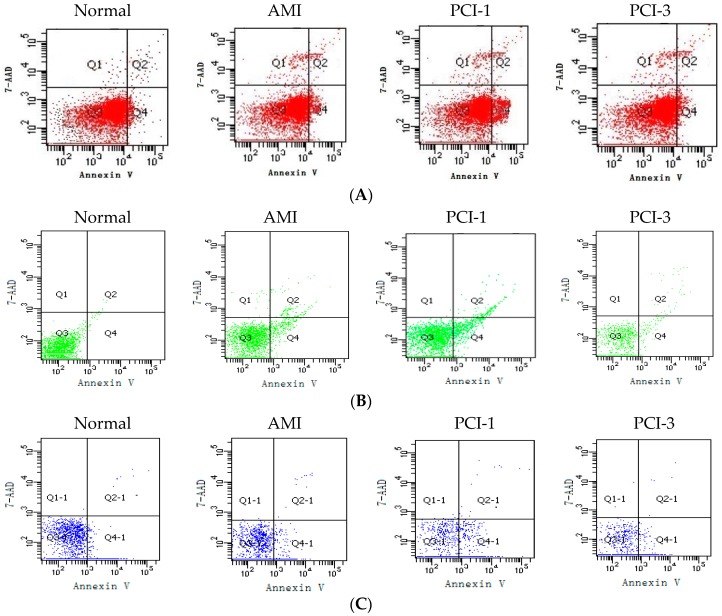

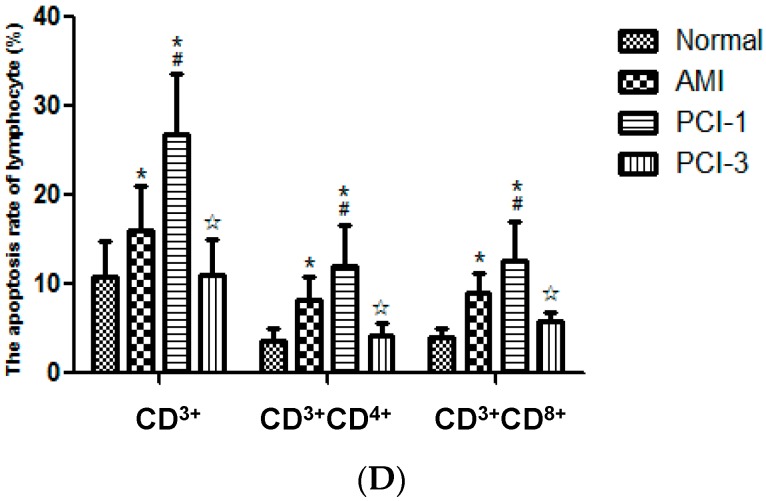
Apoptosis rate of T lymphocytes. FACS results showed that the apoptosis rate of all the T cells of CD^3^^+^ labeled with APC (**A**); CD^3+^CD^4+^ labeled with PE (**B**); and CD^3+^CD^8+^ labeled with APC-Cy7 (**C**) increased at the onset of AMI and PCI-1, especially the CD^3+^ lymphocytes. However, they all returned to normal levels at PCI-3. The apoptosis rate of all these T cells was quantified by densitometry (*n* = 20) (**D**). * *p* < 0.05 vs. Normal group; ^#^ *p* < 0.05 vs. AMI group; ^☆^ *p* < 0.05 vs. PCI-1 group.

Using the Fluorescence activated cell sorting (FACS), we observed the T-cell apoptosis condition. Our results indicated that the apoptosis ratio of CD^3+^ T lymphocytes was about 1.5-fold in AMI, and 3-fold in PCI-1 as much as the control group was (Figure 2A) (*p* < 0.05); the apoptosis ratio of CD^4+^ T lymphocytes was about 2.2-fold in AMI and 3.3-fold in PCI-1, as much as the control group (Figure 2B) (*p* < 0.05); and the apoptosis ratio of CD^8+^ T lymphocytes increased to 2-fold in AMI and to 3.4-fold in PCI-1 more than the control group (*p* < 0.05). They all returned to normal in PCI-3 (Figure 2C).

### 2.5. The Silence Effect of CaSR Small Interfering RNA (siRNA) Transfection

RNA interference (RNAi) refers to the occurrence of specific mRNA degradation mediated by endogenous or exogenous double-stranded RNA (dsRNA), resulting in silencing the expression of a target gene to produce the corresponding functional phenotype missing phenomenon. Small interfering RNA (siRNA) is a small RNA molecule (~21–25 nucleotides) to inspire complementary target mRNA silencing [15].

**Figure 3:**
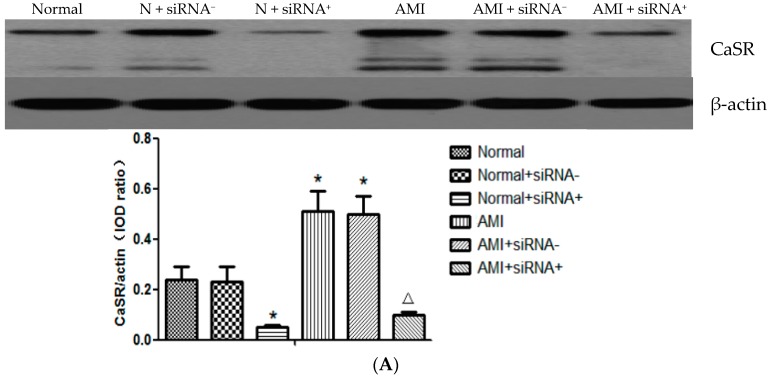

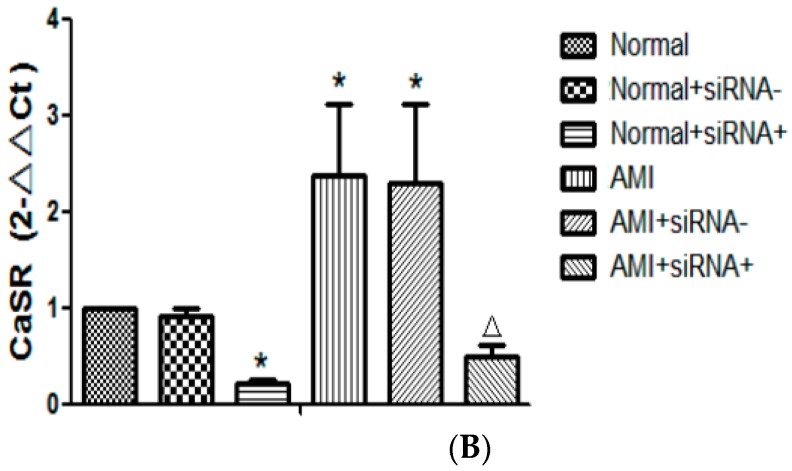
Silence effect of CaSR small interfering RNA (siRNA) transfection. T lymphocytes were transfected with 5 μg positive plasmid or negative plasmid. Protein or mRNA was harvested 24 h post-transfection. Western blot (**A**) and qRT-PCR (**B**) results showed that the positive CaSR siRNA plasmid transfection in T lymphocytes reduced the expressions of CaSR mRNA and protein, which increased remarkably in AMI patients. In addition, negative plasmid of CaSR siRNA had no effect on the CaSR expression. * *p* < 0.05 vs. normal group; ^Δ^ *p* < 0.05 vs. AMI group.

In order to better clarify the role of CaSR in T cells in AMI and PCI, we want to silence the CaSR to observe the effect. Both of the qRT-PCR and Western blot results showed that the expressions of CaSR mRNA and protein in T lymphocytes increased remarkably in AMI patients compared with the normal controls. However, the positive CaSR siRNA plasmid transfection in T lymphocytes reduced the expressions of both CaSR mRNA and protein (*p* < 0.05). In addition, negative plasmid of CaSR siRNA had no effect on the CaSR expression both in the normal and AMI groups (Figure 3).

### 2.6. CaSR siRNA Transfection and NF-κB Pathway Blocker Decreased the Expression of P-p65

**Figure 4:**
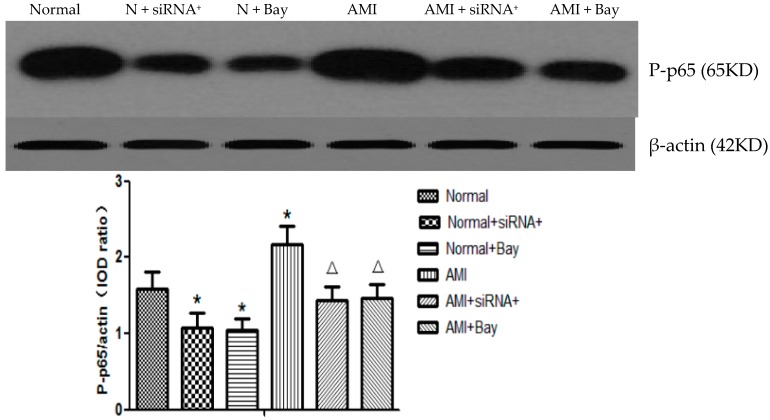
Expression of P-p65 in the T lymphocytes after CaSR siRNA transfection and adding of NF-κB pathway blocker (*n* = 20). The phosphorylation level of p65 protein in T lymphocytes increased greatly in AMI patients. Then, T lymphocytes were transfected by positive CaSR siRNA plasmid for 24 h or cultured by NF-κB pathway blocker Bay-11-7082 (10 mM) for 15 min. In addition, the positive plasmid transfection in T lymphocytes and Bay-11-7082 reduced the expression level both in the normal and AMI groups. * *p* < 0.05 vs. Normal group; ^Δ^ *p* < 0.05 vs. AMI group.

P65 is a subunit of NF-κB, which is a dimmer of members of the Rel proteins family. Activities of NF-κB intracellular signaling proteins often result in the activation of immuno-responsive cells, and the subsequent enhanced production of cytokines [12]. Our results showed that the phosphorylation level of p65 protein in T lymphocytes increased greatly in AMI patients compared with the normal controls. However, the CaSR-positive plasmid transfection in T lymphocytes reduced the expression level both in the normal and AMI groups. The NF-κB pathway blocker Bay-11-7082 had the same effect as positive CaSR siRNA transfection (*p* < 0.05) (Figure 4).

### 2.7. Detection of Cytokines Concentration at the AMI Onset and after PCI

**Figure 5:**
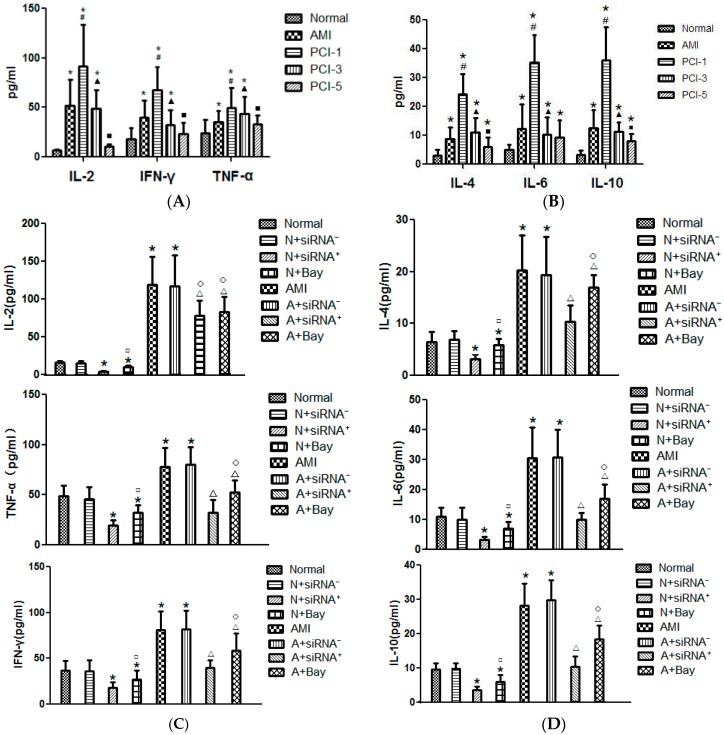
The levels of cytokines in the different stages of AMI and PCI and changed by CaSR siRNA transfection and NF-κB pathway blocker (*n* = 20). The levels of Th-1 type and Th-2 type cytokines in the plasma and the supernatant of cultured T cells were detected by Cytometric Bead Array. (**A**,**B**) Showed that the plasma levels of cytokines in AMI onset and at the different stages of PCI; (**C**,**D**) show the Th-1 and Th-2 type cytokines concentration after T lymphocytes were transfected by CaSR siRNA plasmid for 24 h or cultured by NF-κB pathway blocker Bay-11-7082 (10 mM) for 15 min in the supernatant. * *p* < 0.05 vs. Normal; ^#^ *p* < 0.05 vs. AMI group; ^▲^ *p* < 0.05 vs. PCI-1 group; ^■^ *p* < 0.05 vs. PCI-3 group; ^□^ *p* < 0.05 vs. Normal + siRNA^+^; ^Δ^ *p* < 0.05 vs. AMI; ^◊^ *p* < 0.05 vs. AMI + siRNA^+^.

According to the reports, in response to inflammation in patients with AMI, great deals of cytokines were involved, including Th-1 cytokines and Th-2 cytokines [16,17]. We also know that lymphocytes secrete cytokine to participate in the immunological reaction. In this part, we tested that the levels of both the Th-1 type cytokines and Th-2 type cytokines, such as IL-2, IFN-γ, TNF-α and IL-4, IL-6, IL-10, increased in the plasma at the AMI onset, climbed to the highest level at the PCI-1, and began to decrease from PCI-3. In PCI-5, Th-1 type cytokines dropped to the normal level, but the levels of Th-2 type cytokines were still slightly higher (Figure 5A,B).

### 2.8. CaSR siRNA Transfection and Bay-11-7082 Reduced the Cytokine Secretion

Cytometric Bead Array (CBA) was used to detect the cytokines level in the supernatant of cultured T lymphocytes. In this study, we found that the concentration of Th-1 type and Th-2 type cytokines in AMI group was higher than the normal group (*p* < 0.05). However, positive CaSR siRNA transfection inhibited significantly the all these cytokines secretion. The negative plasmid transfection made no difference in the cytokine release. NF-κB pathway blocker Bay11-7082 also decreased the cytokines level in supernatant, similar with positive CaSR siRNA transfection (*p* < 0.05) (Figure 5C,D).

### 2.9. Positive CaSR siRNA Plasmid and Bay11-7082 Decreased the T Lymphocytes Apoptosis

**Figure 6:**
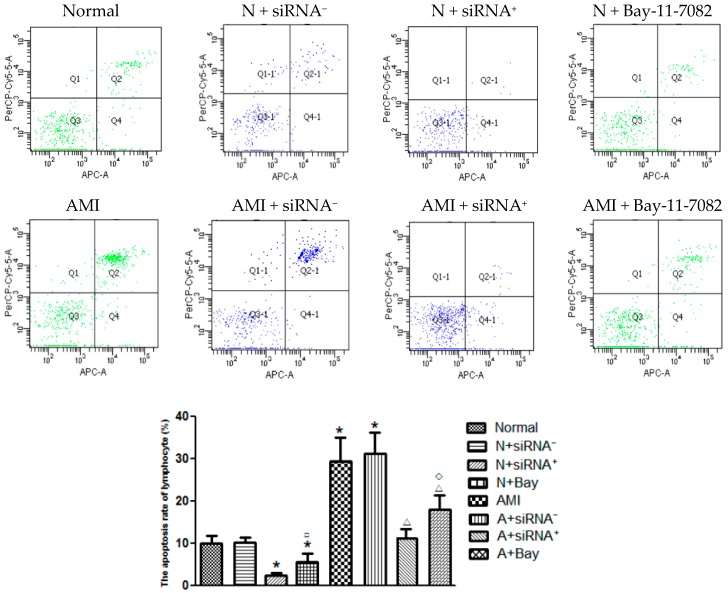
T cells apoptosis rate changed by CaSR siRNA transfection and Bay11-7082 (*n* = 20). The cell apoptosis rate was measured after T lymphocytes were transfected by CaSR siRNA plasmids for 24 h or were cultured by NF-κB pathway blocker Bay-11-7082 (10 mM) for 15 min. The apoptosis rate of all these T cells was quantified by densitometry. * *p* < 0.05 vs. Normal; ^□^ *p* < 0.05 vs. Normal + siRNA^+^; ^Δ^ *p* < 0.05 vs. AMI; ^◊^ *p* < 0.05 vs. AMI + siRNA^+^.

First, we found that the T lymphocytes apoptosis rate in the AMI group was higher than the normal group, and most of the apoptotic cells were in the later stage. Then, we noticed the CaSR siRNA positive- transfection inhibited the cell apoptosis not only in the AMI group but also in the normal group, and most of the apoptotic cells were in the early stage. Negative-transfection lymphocytes had no evident apoptosis appearing whether in the AMI or in the normal group. In addition, NF-κB pathway blocker Bay-11-7082 also decreased the apoptosis degree (*p* < 0.05) (Figure 6). This meant that CaSR and NF-κB pathways affected T lymphocytes apoptosis by interfering in the process of cell period cycle.

## 3. Discussion

AMI is the most dangerous disease that threatens human life. Currently, PCI therapy has become one of the important rescue methods for AMI, but this operation may cause myocardial IR injury and activate neutrophils, lymphocytes, endothelial cells, and platelets to cause the release of a variety of pro-inflammatory and anti-inflammation cytokines, leading to imbalance in the body’s inflammatory response [18,19,20]. We have found that CaSR in T cells was involved in the cytokine secretion and cell apoptosis [11]. However, the CaSR role of T cells on AMI has not been reported. 

During the AMI onset and PCI postoperative, a variety of inflammatory cytokines were released. The changes in cytokines levels take place frequently in infarct tissue. However, the changes may also be detected in the peripheral blood. These cytokines come from many cells. Here, we selected lymphokine as the research object. CD^4+^ T cells include Th-1 and Th-2 subsets according to their secretion of cytokines. Th-1 cells secrete mainly IL-2, IFN-γ and TNF-β. They play an important immune regulation role in mediating organ specific autoimmune disease, organ transplant rejection and anti-infection immunity. Th-2 cells secrete mainly IL-4, IL-5, IL-6, IL-10 and IL-13. They play a decisive role in the humoral immune response. Among them, some research has shown that IL-6 in inflammatory response may mediate artery atherosclerotic plaque vulnerability and burst, and TNF-α may promote atherosclerosis formation [21,22]. The local low TNF-α concentration in myocardial tissue can inhibit myocardial apoptosis, and the higher concentration can promote cell apoptosis and inflammatory reaction [23,24]. It has been shown that plasma concentrations of cytokines produced by Th1 (IFN-γ and IL-2) were significantly higher than those synthesized by Th2 lymphocytes (IL-10) in patients with ACS when compared to patients with stable angina [17]. Another report suggests the high diagnostic value of IL-4 measurements before and immediately after PCI as the correlates of impaired LV dysfunction, whereas only IFN-γ measurement before PCI had a high diagnostic value [16]. Cytokines also were divided into pro-inflammatory and anti-inflammatory cytokines. Many studies have suggested an imbalance between pro-inflammatory and anti-inflammatory markers in acute myocardial infarction (AMI). Both of the pro-inflammatory (IL-6, TNF-α, IL-8) and anti-inflammatory cytokines (IL-4, IL-10, IL-13) constitute a double-edged sword in the inflammation process. Pro-inflammatory cytokines in the early stage are critical to combat with this inflammatory reaction, whereas on the other hand, excessive production can cause tissue and organ damage. A counter-regulatory anti-inflammatory reaction arises in later phases. It was also well known that most AMI patients with PCI postoperative survive the initial phase, but they die during the second stage [12]. In our study, both of the Th-1 and Th-2 type cytokines were released at the AMI onset and climbed to the summit on the PCI-1, then returned to normal gradually, but Th-1 type cytokines declined more quickly than the Th-2 type cytokines. After CaSR siRNA positive-transfection, all of these cytokine levels decreased. In addition, NF-κB pathway blocker Bay-11-7082 also had a similar role. These results indicated that Th-2 cytokines played a greater role than Th-1 in the later stage of AMI reperfusion. According to different roles of cytokines, quantity and time control of these cytokine secretions become more meaningful.

In addition, we also tested the lymphocyte apoptosis condition by FACS and Caspase-12 expression. We found that the CD^3+^, CD^4+^ and CD^8+^ T lymphocyte apoptosis ratios increased at the onset of AMI and PCI-1. Then, on the third day after PCI, all of these indexes returned to basic levels. Caspase-12 is an important component in the apoptosis cascades. We found that the change trend of Caspase-12 in the different stages was consistent with the lymphocyte apoptosis ratio. These results indicated that, in the early ischemia stage, T lymphocytes appeared to make a lot of sacrifices, and, in the later reperfusion stage, T cells recovered gradually. Positive-transfection CaSR inhibited the cells’ apoptosis not only in the AMI group but also in the normal group, and the most apoptotic cells were at the early stage. Bay-11-7082 also decreased the apoptosis degree. In some articles, it has been proven that CaSR activation can promote the cell apoptosis [12,25,26]. Our results further show that both CaSR and NF-κB signaling pathways affected the T lymphocytes apoptosis in AMI and PCI, and CaSR activation can play a role in the cell growth process. 

In recent years, cell signal transduction pathways have become the hotspot of biological science that aim to explore life activities gradually. CaSR can activate a variety of cell signal transduction pathways, and thus play a different physiological function, be involved in cell apoptosis, proliferation, etc. In this experiment, we selected NF-κB transduction pathway as the research target on the basis of previous research. Our team has proved that CaSR in peripheral T lymphocytes contributed to cytokine secretion and apoptosis involved in the NF-κB signaling pathway in sepsis [11,12]. We observed the expressions of CaSR and related channel proteins in peripheral blood T lymphocytes at the AMI onset and in different stages of PCI postoperative, the levels of cytokines, and the apoptosis ratio of T lymphocytes. Firstly, we found that the expression of CaSR and the phosphorylated levels of p65 increased significantly at the AMI onset and PCI postoperative compared the normal group, especially in the PCI-1. They both began to decrease from the PCI-3 and returned to normal in the PCI-5. This shows that the CaSR has some relation to the NF-κB transduction pathway at the AMI onset and progress. Then, we found that CaSR siRNA-positive plasmid transfection decreased the phosphorylated levels of p65 not only in AMI patients but also in normal controls, which further proved that CaSR activation can activate the NF-κB transduction pathway. NF-κB pathway blocker Bay-11-7082 produced the same effect as siRNA transfection. These data provided the evidence that CaSR activation regulated cell physiological functions through NF-κB signaling pathways in AMI and PCI postoperative processes. 

Here, the results speculated that the increased expression and activation of CaSR in the T lymphocytes induced some cytokine secretion in AMI. At the same time, it also induced T-cell apoptosis through the NF-κB signaling pathway.

Certainly, our findings need more cases to be verified due to the large individual differences in humans. Next, we intend to co-culture CaSR-transfected lymphocytes with rat cardiomyocytes under hypoxic conditions imitating myocardial ischemia to further observe the role of CaSR in T lymphocytes on cardiomyocytes. Our study will provide further data on the relationship between AMI and CaSR. Making this relationship clear will provide new effective prevention theories and new targets for drug treatment.

## 4. Materials and Methods

### 4.1. Materials

Anti-CaSR-Ab was purchased from Alomone Labs (Jerusalem, Israel). Anti-Caspase-12-Ab was from Abcam (Cambridge, UK). Anti-P-p65-Ab was from Cell Signaling Technology (Boston, MA, USA). Plasmid was purchased from Life Technologies (Carlsbad, CA, USA). Pacific Blue™ Annexin V Apoptosis Detection Kit with 7-AAD and LEGENDplexTM Multi-analyte Flow Assay Kit were from BioLegend (San Diego, CA, USA). RosetteSep™ Human T cell enrichment cocktail was from Stem Cell Technologies (Vancouver, WA, USA). High Pure RNA Isolation Kit and Transcriptor First Strand cDNA Synthesis Kit was from Roche (Basel, Switzerland). VPA-1002 Human T Cell Nucleofector Kit 25 test was purchased from Lonza (Basel, Switzerland).

### 4.2. Study Population

We prospectively enrolled 60 patients (47 males and 13 females) with AMI (approval was granted by the institutional ethics review board of the Second Affiliated Hospital of Harbin Medical University. No. 2013-064). Informed consent was given for these patients and controls. In addition, the study was performed conform the declaration of Helsinki. They were admitted to the Coronary Heart Disease Care Unit (CCU) of the second affiliated hospital of Harbin Medical University from May 2014 to March 2015. AMI was diagnosed according the new diagnosis standard published by the European Society of Cardiology (ESC) in 2012. The time from acute myocardial infarction onset to admission is about 12 h, and the patients were not taking inflammation inhibiting drugs, such as non-steroidal anti-inflammatory drugs, steroids, and immunosuppressant drugs in the past 3 months. The normal healthy examinations were selected as control, and coronary artery diseases had been ruled out by a number of laboratory tests.

The standard is as follows:

Serum myocardial biochemical markers (mainly serum troponin) increased significantly (at least more than 99% of the normal reference value limit), combining at least one of the following evidences of myocardial ischemia:

(1) The clinical manifestations of myocardial ischemia;
(2) New ischemic electrocardiogram changes, including new ST-T change or left bundle branch block;
(3) Electrocardiogram shows the pathological Q waves;
(4) Imaging shows a new cardiac activity dysfunction or abnormal motion of regional myocardial ventricular wall;
(5) Coronary artery thrombosis is confirmed by coronary angiography examination or autopsy.

### 4.3. Blood Samples

EDTA or heparin anticoagulant peripheral blood (10 mL) was collected from the patients and controls as previous described [11,13]. First, we obtained the lymphocytes by inverted-culture mononuclear leucocytes, which were purified by lymphocyte separation medium (TBD Biological Manufacture Co., Ltd., Tianjin, China). Then, the T lymphocytes were purified by negative selection using the RosetteSep^TM^ human T cell enrichment cocktail (15021, Stem Cell Technologies, Vancouver, BC, Canada) according to the manufacturer’s instructions. The cells were then incubated in the RPMI-1640 medium at 37 °C.

### 4.4. Small Interfering RNA (siRNA) Transfection

T lymphocytes at 8 × 10^6^ were resuspended in 100 μL Nucleofector Solution. Then, they were transfected with 5 μg negative or positive plasmid using Lonza Nucleofector II single nucleus transfection apparatus (Amaxa, Germany). After transfection, cells were recovered 6 h. Protein lysate or mRNA was harvested 24 h post-transfection.

### 4.5. Western Blotting Analysis

As described in a previous paper [12], the T lymphocytes with different conditions were harvested and lysed on ice with protein lysate containing Phenylmethylsulphonylfluoride (PMSF) for 30 min. The protein concentration was determined by using the Bradford protein assay with BSA as standard. Total proteins (20 μg) were subjected to 10% SDS-PAGE and blotted onto polyvinylidene fluoride membrane at 4 V for 20 min. After being blocked in TBS-T containing 5% (*w*/*v*) skimmed milk at 37 °C for 1 h, the membranes were then incubated overnight at 4 °C with various antibodies: anti-CaSR (1:800), anti-Caspase-12 (1:800), and anti-P-p65 (1:1000), respectively, then incubated with anti-IgG antibody conjugated with horseradish peroxidase diluted 1:2000 in TBS-T for 1 h at room temperature. Antibody–antigen complexes were detected by using Western Blue^®^ ECL Plus kit (Beyo, Shanghai, China). Anti-β-actin IgG (Beijing, China) was used (1:400) as the house-keeping internal control. Quantitative comparisons of various proteins under various experimental conditions were performed using a Personal Densitometer™ (Molecular Dynamics, Bio-Rad, Hercules, CA, USA).

### 4.6. Quantitative Real-Time PCR Analysis

Following the manufacturer’s instructions, total RNA was extracted from isolated T lymphocytes and first-strand cDNA was synthesized. The reverse transcription reaction was carried out at 95 °C for 10 min, followed by incubation at 42 °C for 1 h. Five microliters of the RT reaction mixture were used for qPCR amplification in a volume of 50 µL with gene-specific primers designed for the reported sequences:

CaSR (Gene ID: 846): 5′-GTCCAGAAGTCCCTCCCATC-3′ (forward)
                5′-AACCACGCTTTCCTACCCTA-3′ (reverse);
Caspases-12 (Gene ID: 100506742): 5′-TCAACATCCGCAACAAAGAA-3′ (forward)
                    5′-CTCTGGGTGAGCAGCAAACT-3′ (reverse);
β-actin (Gene ID: 60): 5′-AGCGAGCATCCCCCAAAGTT-3′ (forward)
                 5′-GGGCACGAAGGCTCATCATT-3′ (reverse);

qPCR amplification consisted of 40 cycles of denaturation at 95 °C for 15 s, and annealing at 56 °C for 60 s.

### 4.7. Cytokine Analysis by Cytometric Bead Array (CBA)

Blood samples in heparin anticoagulant tube were centrifuged for 10 min (at 2500 r/min) to get the supernatant. The cytokines levels in the supernatant and the medium of cultured T lymphocytes were tested by the commercially available LEGENDplex™ Multi-Analyte Flow Assay Kit according to the manufacturer’s instructions [27].

### 4.8. Apoptosis Detection by FACS

The apoptotic ratio was measured by flow cytometry analysis as previously described [12]. The washing-buffer-washed T lymphocytes were incubated with PE, APC and APC-Cy7, then, with 5 μL Annexin V-Pacific-Blue and 5 μL 7-Amino-Actinomycin (7-AAD) for 15 min at room temperature in the dark according to the manufacturer’s guidelines. Then, the cells were analyzed by flow cytometry (BD LSRF Ortessa, Franklin Lakes, NJ, USA) [13]. 

### 4.9. Statistical Analysis

All the data were obtained from at least three independent experiments and each condition was replicated three times. All values were presented as mean ± Standard deviation. All quantitative data were performed using one-way variance (ANOVA) followed by LSD test. Values of *p* < 0.05 were considered statistically significant.

## 5. Conclusions

Our results demonstrated that calcium-sensing receptor in human peripheral blood T lymphocytes play a role at the AMI onset and progression involving in the NF-κB signaling pathway.
